# (*E*)-3-[(2-Hy­droxy-3-meth­oxy­benzyl­idene)amino]­benzoic acid

**DOI:** 10.1107/S1600536812010549

**Published:** 2012-03-14

**Authors:** Hadi Kargar, Zahra Sharafi, Reza Kia, Safoora Ghelenji, Muhammad Nawaz Tahir

**Affiliations:** a Department of Chemistry, Payame Noor University, PO Box 19395-3697 Tehran, I. R. of IRAN; b Department of Chemistry, Marvdasht Branch, Islamic Azad University, Marvdasht, Iran; c Department of Chemistry, Science and Research Branch, Islamic Azad University, Tehran, Iran; d Department of Chemistry, North Tehran Branch, Islamic Azad University, Tehran, Iran; e Department of Physics, University of Sargodha, Punjab, Pakistan

## Abstract

In the title compound, C_15_H_13_NO_4_, the dihedral angle between the substituted benzene rings is 9.9 (8)°. Part of the mol­ecule (the salicylaldimine segment) is disordered over two sets of sites, with a refined site-occupancy ratio of 0.550 (14):0.450 (14). Intra­molecular O—H⋯N hydrogen bonds form *S*(6) ring motifs. In the crystal, pairs of O—H⋯O hydrogen bonds link mol­ecules into centrosymmetric dimers with *R*_2_^2^(8) ring motifs. The crystal packing also features C—H⋯π inter­actions.

## Related literature

For standard bond lengths, see: Allen *et al.* (1987 ▶). For hydrogen-bond motifs, see: Bernstein *et al.* (1995 ▶). For background to Schiff base ligands and their complexes, see, for example, Kargar *et al.* (2011 ▶, 2012 ▶); Kia *et al.* (2010 ▶).
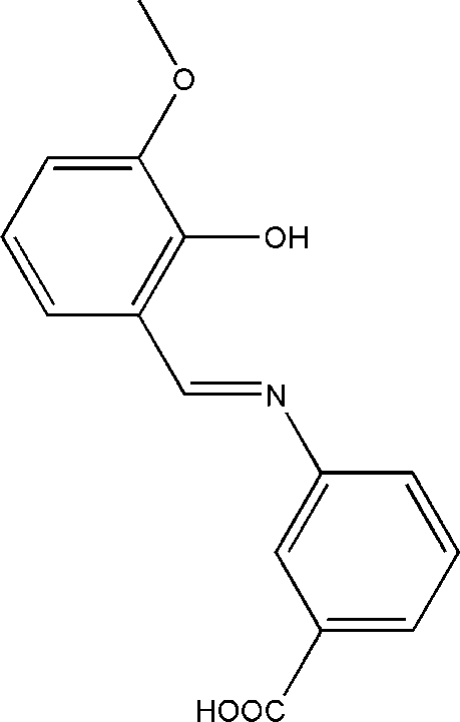


## Experimental

### 

#### Crystal data

C_15_H_13_NO_4_
*M* *_r_* = 271.26
Triclinic, 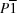

*a* = 5.2738 (9) Å
*b* = 10.978 (2) Å
*c* = 12.084 (2) Å
α = 107.044 (10)°
β = 100.776 (11)°
γ = 97.539 (10)°
*V* = 644.1 (2) Å^3^
*Z* = 2
Mo *K*α radiation
μ = 0.10 mm^−1^
*T* = 296 K
0.19 × 0.12 × 0.09 mm

#### Data collection

Bruker SMART APEXII CCD area-detector diffractometer
Absorption correction: multi-scan (*SADABS*; Bruker, 2005 ▶) *T*_min_ = 0.981, *T*_max_ = 0.991
8775 measured reflections
2308 independent reflections
1253 reflections with *I* > 2σ(*I*)
*R*_int_ = 0.047

#### Refinement

*R*[*F* ^2^ > 2σ(*F* ^2^)] = 0.081
*wR*(*F* ^2^) = 0.258
*S* = 1.05
2308 reflections
279 parameters
405 restraints
H-atom parameters constrained
Δρ_max_ = 0.59 e Å^−3^
Δρ_min_ = −0.28 e Å^−3^

### 

Data collection: *APEX2* (Bruker, 2005 ▶); cell refinement: *SAINT* (Bruker, 2005 ▶); data reduction: *SAINT*; program(s) used to solve structure: *SHELXTL* (Sheldrick, 2008 ▶); program(s) used to refine structure: *SHELXTL*; molecular graphics: *SHELXTL*; software used to prepare material for publication: *SHELXTL* and *PLATON* (Spek, 2009 ▶).

## Supplementary Material

Crystal structure: contains datablock(s) global, I. DOI: 10.1107/S1600536812010549/bq2344sup1.cif

Structure factors: contains datablock(s) I. DOI: 10.1107/S1600536812010549/bq2344Isup2.hkl

Supplementary material file. DOI: 10.1107/S1600536812010549/bq2344Isup3.cml

Additional supplementary materials:  crystallographic information; 3D view; checkCIF report

## Figures and Tables

**Table 1:** Hydrogen-bond geometry (Å, °) *Cg*1 and *Cg*2 are the centroids of the C9–C14 and C9*A*–C14*A* rings, respectively.

*D*—H⋯*A*	*D*—H	H⋯*A*	*D*⋯*A*	*D*—H⋯*A*
O2—H2⋯O1^i^	0.96	1.69	2.638 (4)	169
O3—H3⋯N1	0.82	1.92	2.64 (2)	147
C15*A*—H15*E*⋯*Cg*1^ii^	0.96	2.90	3.757 (11)	139
C15*A*—H15*E*⋯*Cg*2^ii^	0.96	2.83	3.680 (12)	139

Symmetry codes: (i) 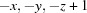; (ii) 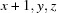.

**Fig. 1.**
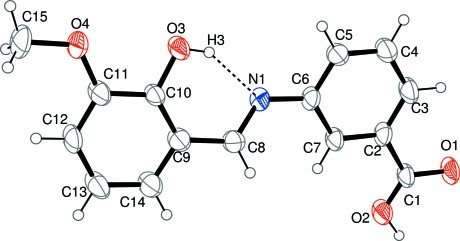
The ORTEP plot of the title compound, showing 40% probability displacement ellipsoids and the atomic numbering. The major component of the disordered part was used. The dashed line shows the intramolecular H-bonding.

**Fig. 2.**
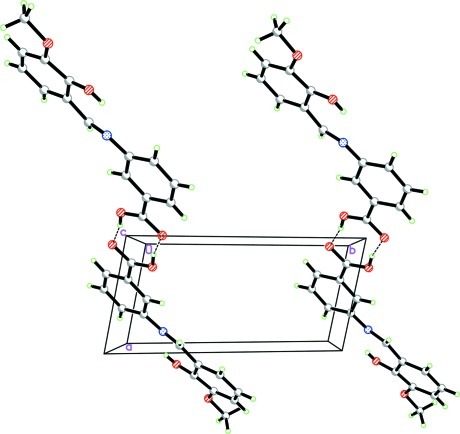
The packing diagram of the title compound viewed down the c-axis showing linking of dimers with R22(8) ring motifs. Only the hydrogen atoms involved the H-bonding and the major component are shown.

## supplementary crystallographic information

### Comment

In continuation of our work on the crystal structure of Schiff base ligands (Kargar *et al.,* 2011; Kia *et al.,* 2010; Kargar *et al.,* 2012), we determined the X-ray structure of the title compound.

The asymmetric unit of the title compound, Fig. 1, comprises a potentially bidentate N,O-donor Schiff base ligand. The bond lengths (Allen *et al.,* 1987) and angles are within the normal ranges.

The intramolecular O—H···N hydrogen bonds make S(6) ring motifs (Bernstein *et al.*, 1995). The dihedral angle between the substituted benzene rings is 9.9 (8)°. Pairs of the centrosymmetric intermolecular O—H···O hydrogen bonds link molecules into dimers with *R^2^_2_(8)* ring motifs (Fig. 2). A part of the molecule was disordered over two positions with a refined site occupancy ratio 0.55 (1)/0.45 (1). The crystal packing was further stabilized by the intermolecular C—H···π interactions (Table 2).

### Experimental

The title compound was synthesized by adding 3-methoxyosalicylaldehyde (2 mmol) to a solution of 3-carboxyaniline (2 mmol) in ethanol (30 ml). The mixture was refluxed with stirring for half an hour. The resultant solution was filtered. Pale yellow single crystals of the title compound suitable for *X*-ray structure determination were recrystallized from ethanol by slow evaporation of the solvents at room temperature over several days.

### Refinement

The O-bound hydrogen atoms were positioned by a rotating O—H group model and constrained to the parent atoms with U_iso_ (H) = 1.5 U_eq_ (O). The rest of the hydrogen atoms were positioned geometrically with C—H = 0.93-0.97 Å and included in a riding model approximation with U_iso_ (H) = 1.2 or 1.5 U_eq_ (C). A rotating group model was applied to the methyl group. Since the crystal was very small and not optimal for diffraction the Data/Parameter ratio was not good. The similarity restraints (SIMU, DELU, and SAME) were applied to model the disorder.

### Crystal data

**Table:**

C_15_H_13_NO_4_	*Z* = 2
*M**_r_* = 271.26	*F*(000) = 284
Triclinic, *P*1	*D*_x_ = 1.399 Mg m^−^^3^
Hall symbol: -P 1	Mo *K*α radiation, λ = 0.71073 Å
*a* = 5.2738 (9) Å	Cell parameters from 1456 reflections
*b* = 10.978 (2) Å	θ = 2.8–28.8°
*c* = 12.084 (2) Å	µ = 0.10 mm^−^^1^
α = 107.044 (10)°	*T* = 296 K
β = 100.776 (11)°	Block, pale-yellow
γ = 97.539 (10)°	0.19 × 0.12 × 0.09 mm
*V* = 644.1 (2) Å^3^	

### Data collection

**Table:**

Bruker SMART APEXII CCD area-detector diffractometer	2308 independent reflections
Radiation source: fine-focus sealed tube	1253 reflections with *I* > 2σ(*I*)
Graphite monochromator	*R*_int_ = 0.047
φ and ω scans	θ_max_ = 25.3°, θ_min_ = 2.2°
Absorption correction: multi-scan (*SADABS*; Bruker, 2005)	*h* = −6→6
*T*_min_ = 0.981, *T*_max_ = 0.991	*k* = −13→13
8775 measured reflections	*l* = −14→14

### Refinement

**Table:**

Refinement on *F*^2^	Primary atom site location: structure-invariant direct methods
Least-squares matrix: full	Secondary atom site location: difference Fourier map
*R*[*F*^2^ > 2σ(*F*^2^)] = 0.081	Hydrogen site location: inferred from neighbouring sites
*wR*(*F*^2^) = 0.258	H-atom parameters constrained
*S* = 1.05	*w* = 1/[σ^2^(*F*_o_^2^) + (0.1461*P*)^2^ + 0.0613*P*] where *P* = (*F*_o_^2^ + 2*F*_c_^2^)/3
2308 reflections	(Δ/σ)_max_ < 0.001
279 parameters	Δρ_max_ = 0.59 e Å^−^^3^
405 restraints	Δρ_min_ = −0.28 e Å^−^^3^

### Special details

**Table:**

Geometry. All esds (except the esd in the dihedral angle between two l.s. planes) are estimated using the full covariance matrix. The cell esds are taken into account individually in the estimation of esds in distances, angles and torsion angles; correlations between esds in cell parameters are only used when they are defined by crystal symmetry. An approximate (isotropic) treatment of cell esds is used for estimating esds involving l.s. planes.
Refinement. Refinement of F^2^ against ALL reflections. The weighted R-factor wR and goodness of fit S are based on F^2^, conventional R-factors R are based on F, with F set to zero for negative F^2^. The threshold expression of F^2^ > 2sigma(F^2^) is used only for calculating R-factors(gt) etc. and is not relevant to the choice of reflections for refinement. R-factors based on F^2^ are statistically about twice as large as those based on F, and R- factors based on ALL data will be even larger.

### Fractional atomic coordinates and isotropic or equivalent isotropic displacement parameters (Å^2^)

**Table:**

	*x*	*y*	*z*	*U*_iso_*/*U*_eq_	Occ. (<1)
C1	0.2047 (6)	−0.0156 (3)	0.3825 (3)	0.0503 (9)	
C2	0.3579 (6)	−0.0263 (3)	0.2901 (3)	0.0469 (8)	
C3	0.3065 (7)	−0.1382 (3)	0.1929 (3)	0.0605 (10)	
H3A	0.1755	−0.2078	0.1850	0.073*	
C4	0.4508 (7)	−0.1461 (3)	0.1075 (3)	0.0659 (11)	
H4A	0.4174	−0.2212	0.0421	0.079*	
C5	0.6418 (7)	−0.0437 (3)	0.1192 (3)	0.0537 (9)	
H5A	0.7389	−0.0505	0.0616	0.064*	
C6	0.6948 (6)	0.0696 (3)	0.2142 (3)	0.0461 (8)	
C7	0.5508 (6)	0.0785 (3)	0.3021 (3)	0.0472 (8)	
H7A	0.5847	0.1537	0.3675	0.057*	
O1	0.0350 (5)	−0.1160 (2)	0.3720 (2)	0.0663 (8)	
O2	0.2431 (5)	0.0872 (2)	0.4652 (2)	0.0699 (8)	
H2	0.1331	0.0870	0.5202	0.105*	
N1	0.868 (3)	0.1754 (14)	0.215 (2)	0.038 (3)	0.550 (14)
C8	0.997 (2)	0.2711 (10)	0.3133 (11)	0.051 (3)	0.550 (14)
H8A	0.9624	0.2715	0.3861	0.061*	0.550 (14)
C9	1.192 (2)	0.3752 (9)	0.3088 (10)	0.054 (4)	0.550 (14)
C10	1.307 (8)	0.363 (2)	0.213 (3)	0.044 (5)	0.550 (14)
C11	1.473 (4)	0.4714 (14)	0.2060 (17)	0.058 (5)	0.550 (14)
C12	1.552 (7)	0.5805 (19)	0.305 (3)	0.062 (6)	0.550 (14)
H12A	1.6724	0.6497	0.3034	0.075*	0.550 (14)
C13	1.459 (2)	0.5914 (9)	0.4063 (10)	0.075 (3)	0.550 (14)
H13A	1.5152	0.6665	0.4722	0.090*	0.550 (14)
C14	1.281 (2)	0.4882 (9)	0.4072 (9)	0.073 (3)	0.550 (14)
H14A	1.2184	0.4940	0.4752	0.088*	0.550 (14)
C15	1.752 (2)	0.5546 (13)	0.0982 (14)	0.080 (4)	0.550 (14)
H15A	1.8146	0.5250	0.0272	0.121*	0.550 (14)
H15B	1.8972	0.5807	0.1667	0.121*	0.550 (14)
H15C	1.6696	0.6273	0.0964	0.121*	0.550 (14)
O3	1.207 (2)	0.2641 (12)	0.1078 (10)	0.065 (3)	0.550 (14)
H3	1.0896	0.2127	0.1154	0.097*	0.550 (14)
O4	1.565 (2)	0.4519 (10)	0.1044 (8)	0.080 (3)	0.550 (14)
N1A	0.929 (4)	0.1671 (17)	0.222 (3)	0.038 (3)	0.450 (14)
C8A	0.922 (2)	0.2889 (10)	0.2830 (11)	0.038 (3)	0.450 (14)
H8AA	0.7868	0.3062	0.3209	0.045*	0.450 (14)
C9A	1.128 (3)	0.3943 (10)	0.2899 (12)	0.044 (3)	0.450 (14)
C10A	1.287 (10)	0.374 (2)	0.210 (4)	0.041 (4)	0.450 (14)
C11A	1.503 (5)	0.4729 (15)	0.2231 (19)	0.044 (3)	0.450 (14)
C12A	1.521 (9)	0.595 (2)	0.300 (3)	0.062 (5)	0.450 (14)
H12B	1.6540	0.6625	0.3042	0.074*	0.450 (14)
C13A	1.344 (3)	0.6185 (9)	0.3695 (10)	0.061 (3)	0.450 (14)
H13B	1.3546	0.7023	0.4197	0.073*	0.450 (14)
C14A	1.151 (2)	0.5199 (7)	0.3662 (9)	0.057 (3)	0.450 (14)
H14B	1.0346	0.5371	0.4153	0.068*	0.450 (14)
C15A	1.854 (3)	0.5432 (16)	0.1425 (16)	0.077 (4)	0.450 (14)
H15D	1.9220	0.5133	0.0732	0.115*	0.450 (14)
H15E	1.9947	0.5697	0.2127	0.115*	0.450 (14)
H15F	1.7723	0.6156	0.1387	0.115*	0.450 (14)
O3A	1.295 (3)	0.2529 (14)	0.1380 (15)	0.062 (4)	0.450 (14)
H3B	1.1719	0.1997	0.1393	0.093*	0.450 (14)
O4A	1.661 (2)	0.4394 (11)	0.1466 (11)	0.062 (3)	0.450 (14)

### Atomic displacement parameters (Å^2^)

**Table:**

	*U*^11^	*U*^22^	*U*^33^	*U*^12^	*U*^13^	*U*^23^
C1	0.049 (2)	0.0358 (17)	0.064 (2)	−0.0037 (15)	0.0263 (17)	0.0113 (16)
C2	0.0416 (18)	0.0415 (17)	0.061 (2)	0.0011 (15)	0.0226 (16)	0.0186 (16)
C3	0.058 (2)	0.0408 (18)	0.076 (2)	−0.0084 (16)	0.029 (2)	0.0086 (17)
C4	0.074 (3)	0.0459 (19)	0.069 (2)	−0.0080 (18)	0.035 (2)	0.0019 (17)
C5	0.054 (2)	0.0481 (19)	0.054 (2)	−0.0007 (16)	0.0273 (17)	0.0053 (16)
C6	0.0400 (18)	0.0430 (17)	0.057 (2)	0.0004 (14)	0.0226 (16)	0.0162 (15)
C7	0.0413 (18)	0.0389 (17)	0.059 (2)	−0.0023 (14)	0.0248 (16)	0.0097 (15)
O1	0.0638 (16)	0.0449 (13)	0.0852 (18)	−0.0150 (11)	0.0389 (14)	0.0117 (12)
O2	0.0772 (18)	0.0519 (15)	0.0748 (18)	−0.0139 (13)	0.0489 (14)	0.0046 (13)
N1	0.017 (7)	0.044 (2)	0.053 (3)	0.003 (3)	0.015 (5)	0.013 (2)
C8	0.045 (6)	0.055 (5)	0.046 (5)	−0.002 (4)	0.012 (4)	0.010 (4)
C9	0.048 (6)	0.050 (4)	0.057 (5)	0.001 (4)	0.025 (5)	0.003 (4)
C10	0.038 (8)	0.038 (6)	0.047 (5)	−0.002 (6)	0.014 (5)	0.002 (5)
C11	0.038 (6)	0.054 (5)	0.065 (7)	−0.007 (5)	0.025 (6)	−0.004 (5)
C12	0.045 (8)	0.051 (7)	0.080 (7)	−0.014 (7)	0.027 (6)	0.007 (6)
C13	0.058 (6)	0.057 (5)	0.082 (6)	−0.015 (4)	0.037 (5)	−0.018 (4)
C14	0.063 (6)	0.068 (5)	0.070 (5)	−0.015 (4)	0.038 (5)	−0.005 (4)
C15	0.057 (7)	0.079 (6)	0.105 (10)	−0.012 (5)	0.043 (7)	0.027 (6)
O3	0.057 (7)	0.052 (5)	0.067 (5)	−0.018 (4)	0.037 (5)	−0.009 (4)
O4	0.073 (6)	0.073 (4)	0.075 (5)	−0.030 (4)	0.040 (4)	0.004 (4)
N1A	0.017 (7)	0.044 (2)	0.053 (3)	0.003 (3)	0.015 (5)	0.013 (2)
C8A	0.027 (5)	0.042 (4)	0.047 (7)	0.008 (3)	0.021 (4)	0.010 (4)
C9A	0.040 (6)	0.034 (4)	0.057 (6)	0.000 (4)	0.020 (5)	0.013 (4)
C10A	0.030 (7)	0.033 (5)	0.062 (8)	0.004 (5)	0.021 (6)	0.013 (5)
C11A	0.042 (7)	0.042 (5)	0.054 (7)	0.001 (4)	0.017 (5)	0.023 (5)
C12A	0.055 (10)	0.044 (6)	0.077 (8)	−0.006 (6)	0.020 (7)	0.009 (6)
C13A	0.071 (7)	0.036 (4)	0.066 (6)	−0.004 (4)	0.022 (5)	0.005 (4)
C14A	0.060 (6)	0.039 (4)	0.070 (6)	0.006 (4)	0.028 (5)	0.010 (4)
C15A	0.067 (9)	0.069 (7)	0.099 (11)	−0.017 (7)	0.035 (7)	0.039 (7)
O3A	0.059 (8)	0.038 (4)	0.084 (8)	−0.002 (5)	0.041 (6)	0.002 (5)
O4A	0.050 (5)	0.064 (4)	0.076 (7)	−0.009 (4)	0.039 (5)	0.024 (4)

### Geometric parameters (Å, º)

**Table:**

C1—O2	1.232 (4)	C13—C14	1.378 (9)
C1—O1	1.284 (4)	C13—H13A	0.9300
C1—C2	1.482 (5)	C14—H14A	0.9300
C2—C3	1.382 (4)	C15—O4	1.426 (9)
C2—C7	1.385 (4)	C15—H15A	0.9600
C3—C4	1.382 (5)	C15—H15B	0.9600
C3—H3A	0.9300	C15—H15C	0.9600
C4—C5	1.361 (4)	O3—H3	0.8200
C4—H4A	0.9300	N1A—C8A	1.333 (13)
C5—C6	1.376 (4)	C8A—C9A	1.450 (10)
C5—H5A	0.9300	C8A—H8AA	0.9300
C6—N1	1.38 (2)	C9A—C10A	1.382 (12)
C6—C7	1.405 (4)	C9A—C14A	1.391 (10)
C6—N1A	1.49 (3)	C10A—O3A	1.368 (19)
C7—H7A	0.9300	C10A—C11A	1.419 (14)
O2—H2	0.9612	C11A—O4A	1.362 (12)
N1—C8	1.329 (13)	C11A—C12A	1.367 (16)
C8—C9	1.454 (9)	C12A—C13A	1.374 (17)
C8—H8A	0.9300	C12A—H12B	0.9300
C9—C10	1.383 (11)	C13A—C14A	1.370 (10)
C9—C14	1.395 (9)	C13A—H13B	0.9300
C10—O3	1.365 (18)	C14A—H14B	0.9300
C10—C11	1.418 (13)	C15A—O4A	1.443 (10)
C11—C12	1.370 (15)	C15A—H15D	0.9600
C11—O4	1.373 (11)	C15A—H15E	0.9600
C12—C13	1.384 (16)	C15A—H15F	0.9600
C12—H12A	0.9300	O3A—H3B	0.8200
			
O2—C1—O1	122.7 (3)	C11—C12—H12A	118.8
O2—C1—C2	119.7 (3)	C13—C12—H12A	118.8
O1—C1—C2	117.6 (3)	C14—C13—C12	118.3 (8)
C3—C2—C7	120.6 (3)	C14—C13—H13A	120.9
C3—C2—C1	120.5 (3)	C12—C13—H13A	120.9
C7—C2—C1	118.9 (3)	C13—C14—C9	121.8 (7)
C2—C3—C4	119.7 (3)	C13—C14—H14A	119.1
C2—C3—H3A	120.2	C9—C14—H14A	119.1
C4—C3—H3A	120.2	C11—O4—C15	118.0 (9)
C5—C4—C3	120.0 (3)	C8A—N1A—C6	113.9 (18)
C5—C4—H4A	120.0	N1A—C8A—C9A	119.6 (14)
C3—C4—H4A	120.0	N1A—C8A—H8AA	120.2
C4—C5—C6	121.7 (3)	C9A—C8A—H8AA	120.2
C4—C5—H5A	119.2	C10A—C9A—C14A	118.6 (10)
C6—C5—H5A	119.2	C10A—C9A—C8A	119.5 (9)
C5—C6—N1	119.2 (10)	C14A—C9A—C8A	121.5 (10)
C5—C6—C7	118.9 (3)	O3A—C10A—C9A	123.2 (14)
N1—C6—C7	121.5 (9)	O3A—C10A—C11A	115 (2)
C5—C6—N1A	115.4 (10)	C9A—C10A—C11A	119.5 (10)
N1—C6—N1A	13.2 (17)	O4A—C11A—C12A	125.1 (11)
C7—C6—N1A	125.2 (11)	O4A—C11A—C10A	115.2 (10)
C2—C7—C6	119.2 (3)	C12A—C11A—C10A	119.3 (16)
C2—C7—H7A	120.4	C11A—C12A—C13A	120.0 (13)
C6—C7—H7A	120.4	C11A—C12A—H12B	120.0
C1—O2—H2	114.7	C13A—C12A—H12B	120.0
C8—N1—C6	123 (2)	C14A—C13A—C12A	120.9 (10)
N1—C8—C9	120.7 (14)	C14A—C13A—H13B	119.5
N1—C8—H8A	119.6	C12A—C13A—H13B	119.5
C9—C8—H8A	119.6	C13A—C14A—C9A	120.4 (9)
C10—C9—C14	118.3 (8)	C13A—C14A—H14B	119.8
C10—C9—C8	122.2 (8)	C9A—C14A—H14B	119.8
C14—C9—C8	119.4 (8)	O4A—C15A—H15D	109.5
O3—C10—C9	121.7 (12)	O4A—C15A—H15E	109.5
O3—C10—C11	115.0 (19)	H15D—C15A—H15E	109.5
C9—C10—C11	120.3 (8)	O4A—C15A—H15F	109.5
C12—C11—O4	125.4 (9)	H15D—C15A—H15F	109.5
C12—C11—C10	118.0 (14)	H15E—C15A—H15F	109.5
O4—C11—C10	116.1 (9)	C10A—O3A—H3B	109.5
C11—C12—C13	122.4 (10)	C11A—O4A—C15A	116.8 (11)
			
O2—C1—C2—C3	−176.1 (3)	O4—C11—C12—C13	−177 (3)
O1—C1—C2—C3	3.8 (5)	C10—C11—C12—C13	−5 (7)
O2—C1—C2—C7	3.1 (5)	C11—C12—C13—C14	0 (5)
O1—C1—C2—C7	−177.1 (3)	C12—C13—C14—C9	−1 (3)
C7—C2—C3—C4	0.6 (5)	C10—C9—C14—C13	7 (3)
C1—C2—C3—C4	179.8 (3)	C8—C9—C14—C13	−177.9 (9)
C2—C3—C4—C5	−0.2 (6)	C12—C11—O4—C15	−2 (4)
C3—C4—C5—C6	−0.7 (6)	C10—C11—O4—C15	−174 (3)
C4—C5—C6—N1	−172.3 (11)	C5—C6—N1A—C8A	156.3 (18)
C4—C5—C6—C7	1.2 (5)	N1—C6—N1A—C8A	46 (7)
C4—C5—C6—N1A	173.4 (14)	C7—C6—N1A—C8A	−32 (3)
C3—C2—C7—C6	−0.1 (5)	C6—N1A—C8A—C9A	−175.4 (16)
C1—C2—C7—C6	−179.3 (3)	N1A—C8A—C9A—C10A	17 (4)
C5—C6—C7—C2	−0.7 (5)	N1A—C8A—C9A—C14A	−170.5 (19)
N1—C6—C7—C2	172.6 (11)	C14A—C9A—C10A—O3A	175 (4)
N1A—C6—C7—C2	−172.2 (14)	C8A—C9A—C10A—O3A	−13 (7)
C5—C6—N1—C8	−157.2 (13)	C14A—C9A—C10A—C11A	13 (7)
C7—C6—N1—C8	29 (2)	C8A—C9A—C10A—C11A	−174 (4)
N1A—C6—N1—C8	−81 (10)	O3A—C10A—C11A—O4A	11 (6)
C6—N1—C8—C9	175.3 (12)	C9A—C10A—C11A—O4A	174 (4)
N1—C8—C9—C10	−19 (3)	O3A—C10A—C11A—C12A	−176 (4)
N1—C8—C9—C14	165.7 (13)	C9A—C10A—C11A—C12A	−13 (8)
C14—C9—C10—O3	−171 (3)	O4A—C11A—C12A—C13A	177 (3)
C8—C9—C10—O3	14 (5)	C10A—C11A—C12A—C13A	5 (8)
C14—C9—C10—C11	−12 (5)	C11A—C12A—C13A—C14A	2 (7)
C8—C9—C10—C11	173 (3)	C12A—C13A—C14A—C9A	−1 (3)
O3—C10—C11—C12	172 (4)	C10A—C9A—C14A—C13A	−6 (4)
C9—C10—C11—C12	11 (7)	C8A—C9A—C14A—C13A	−178.7 (11)
O3—C10—C11—O4	−16 (5)	C12A—C11A—O4A—C15A	−3 (5)
C9—C10—C11—O4	−176 (3)	C10A—C11A—O4A—C15A	169 (4)

### Hydrogen-bond geometry (Å, º)

Cg1 and Cg2 are the centroids of the C9–C14 and C9A–C14A rings, respectively.

**Table:**

*D*—H···*A*	*D*—H	H···*A*	*D*···*A*	*D*—H···*A*
O2—H2···O1^i^	0.96	1.69	2.638 (4)	169
O3—H3···N1	0.82	1.92	2.64 (2)	147
C15*A*—H15*E*···*Cg*1^ii^	0.96	2.90	3.757 (11)	139
C15*A*—H15*E*···*Cg*2^ii^	0.96	2.83	3.680 (12)	139

Symmetry codes: (i) −*x*, −*y*, −*z*+1; (ii) *x*+1, *y*, *z*.
